# Infants’ brain responses to pupillary changes in others are affected by race

**DOI:** 10.1038/s41598-019-40661-z

**Published:** 2019-03-13

**Authors:** Caroline M. Kelsey, Kathleen M. Krol, Mariska E. Kret, Tobias Grossmann

**Affiliations:** 1 Department of Psychology, University of Virginia, Charlottesville, VA USA; 2 Institute of Psychology, Cognitive Psychology Unit, Leiden University, Leiden, The Netherlands; 3 Max Planck Institute for Human Cognitive and Brain Sciences, Leipzig, Germany; 4 Leiden University, Leiden Institute for Brain and Cognition (LIBC), Leiden, The Netherlands

## Abstract

Sensitive responding to eye cues plays a key role during human social interactions. Observed changes in pupillary size provide a range of socially-relevant information including cues regarding a person’s emotional and arousal states. Recently, infants have been found to mimic observed pupillary changes in others, instantiating a foundational mechanism for eye-based social communication. Among adults, perception of pupillary changes is affected by race. Here, we examined whether and how race impacts the neural processing of others’ pupillary changes in early ontogeny. We measured 9-month-old infants’ brain responses to dilating and constricting pupils in the context of viewing own-race and other-race eyes using functional near-infrared spectroscopy (fNIRS). Our results show that only when responding to own-race eyes, infants’ brains distinguished between changes in pupillary size. Specifically, infants showed enhanced responses in the right superior temporal cortex when observing own-race pupil dilation. Moreover, when processing other-race pupillary changes, infants recruited the dorsolateral prefrontal cortex, a brain region linked to cognitive control functions. These findings suggest that, early in development, the fundamental process of responding to pupillary changes is impacted by race and interracial interactions may afford greater cognitive control or effort. This critically informs our understanding of the early origins of responding to pupillary signals in others and further highlights the impact of race on the processing of social signals.

## Introduction

The ability to detect and respond to information from the eyes is an early developing capacity that is considered a foundational feature of human social cognition in infancy^1,2^. During face-to-face social interactions, information regarding a person’s attentional, emotional, and mental state can be gleaned from the eye region and from the pupillary state^3–5^. By adulthood, a preference develops for individuals with larger pupils and pupil dilation is recognized as a signal for positive affect^4,6^. Though this preference has been shown to emerge in early adulthood, much less is known about how infants perceive and respond to pupillary cues. Recent work shows that early in ontogeny humans already differentially respond to changes in others’ pupil size (diameter). Specifically, infants as young as 4 months of age display greater pupil dilation when viewing photographs of dilated eyes, suggesting pupil dilation mimicry^7^. Similarly, infants 6 to 9 months of age show greater pupil dilation when viewing larger compared to smaller schematic eyes^7,8^. Considering that changes in pupil size are closely tied to changes in physiological arousal controlled by the autonomic nervous system, it is possible that the coordination of arousal between social partners facilitates pupil mimicry or alternatively that pupil mimicry facilitates the unconscious coordination of arousal between social partners^6–10^. Regardless of the exact directionality of how pupil mimicry and the coordination of arousal between social partners are linked, responding to others’ pupillary changes has been argued to play an important role in guiding and impacting interpersonal contact^11^. More specifically, from a developmental perspective, although the aforementioned studies with infants attest that pupil dilation mimicry exists early in ontogeny, many question remain regarding the nature of infants’ sensitivity to others’ pupillary changes.

Importantly, research with adults shows that pupil mimicry between social partners depends on race. Specifically, in Kret, Fischer, and De Dreu’s study, adults displayed greater pupil dilation mimicry for own-race members than for other-race members. Moreover, this study found that adults trusted others with dilating pupils more, but this effect on trust was only seen when responding to own-race partners’ eyes^11^. These findings emphasize that the relation between pupillary cues and overt, social behavior are impacted by race in adults. Moreover, these results are interpreted as adults being more sensitive to social signals in the context of their own-race due to biases that have developed over time. Yet, it is unknown whether just lack of familiarity, and not the protracted development of racial biases, influence the detection of pupillary cues. The second goal of the current study, therefore, was to examine the impact of race on infants’ responses to observed pupillary changes. Already by 3 months of age infants have been shown to visually prefer own-race over other-race faces^12^. By around 9 months of age, perceptual narrowing to familiar race faces occurs in infants, resulting in impeded identity and emotion recognition from other-race faces^13,14^. Furthermore, between 4 and 9 months of age, infants’ fixations on internal facial features decrease only in response to other-race faces but not to own-race faces^15^. Based on previous work showing that by around 9 months of age infants’ processing of social information (identifying individuals and emotion discrimination) is intact for own-race faces but impeded for other-race faces^13,14^, we decided to study infants at 9 months of age. Given the role of attentional processes and experience, we also assessed how attention to stimuli and other-race exposure affects the response to own- and other-race pupillary cues.

It is important to note that infants at this age do show a visual preference for own-race faces but do not yet show racial biases in their overt social behavior. More specifically, prior work has shown that 10-month-old infants were equally likely to take a toy from an own-race when compared to an other-race member^16^. In this study, 24-month-old infants were also found to be equally likely to give a toy to an own-race when compared to an other-race member, and it was not until 5 years of age that children displayed an overt bias (i.e., children were more prosocial towards an own-race member as compared to an other-race member)^16^.

One way to further understand how infants respond to pupillary signals is to measure the brain processes involved in infants’ detection of pupillary changes in others. Thus, one goal of the current study was to examine the neural correlates of processing pupillary changes in others by measuring localized cortical brain responses using functional near-infrared spectroscopy (fNIRS). Prior work with adults using functional magnetic resonance imaging (fMRI) shows that particularly the right superior temporal sulcus, a region implicated in processing a wide array of dynamic social information from faces, including emotional and gaze cues^17^, also responds to changes in pupil size^18,19^. In this context, it is important to emphasize that, similar to adults, there is evidence from previous studies with infants showing that the superior temporal cortex, especially in the right hemisphere, is involved in eye gaze and emotion processing from early in infancy^20,21^. On the basis of the abovementioned studies examining the neural correlates of processing pupillary changes in adults and given the neural sensitivity to dynamic eye gaze and emotional cues in the superior temporal cortex previously demonstrated in infants^20,22,23^, we hypothesized that observed changes in pupil size will result in differential brain responses in infants’ superior temporal cortex (STC). Furthermore, considering the lateralization of STC responses reported in infants and adults^20,21,24^, we decided to test for brain response lateralization by including hemisphere (left and right) as a factor in our fNIRS analyses.

With respect to the brain process involved when encountering faces of other-race individuals, our use of fNIRS as the neuroimaging method with infants limits our investigation to cortical brain regions and does not allow us to image responses from subcortical brain structures such as the amygdala^25^. One cortical region that is commonly engaged during interracial interactions in adults is the dorsolateral prefrontal cortex (dlPFC)^26^. This region is generally implicated in cognitive control and its enhanced activity during interracial contact is thought to reflect both self-regulatory processes, involved in attenuating racial bias during interracial encounters^26,27^, and increased effort, due to lack of familiarity and exposure to other-race individuals^28^. Although the prefrontal cortex is known to show protracted structural and functional development extending well beyond infancy, evidence is mounting that prefrontal regions are functionally active from earlier than previously thought^29^, and may specifically be involved in cognitive processes reflecting novelty detection during infancy^30^. We therefore hypothesized that 9-month-old infants show enhanced responses in dlPFC when processing other-race stimuli.

Taken together, the current study was designed to shed light on the nature and early development of sensitivity to pupillary cues by examining the brain processes involved in and the influence of racial context on infants’ responses to observed pupillary changes in social partners. In order to achieve this, we adapted an experimental paradigm previously used with adults^11,31^. We presented 9-month-old infants with own-race and other-race eyes that were either dilating or constricting, while measuring their brain responses in frontal and temporal cortices using fNIRS. Specifically, we used Japanese, Asian, eyes as the other-race comparison. Asian eyes were selected as the experimental out-group because of previous adult work that has used this comparison and shown differences in behavioral response to own- versus other-race stimuli^11^. In addition, previous research with infants has shown that by 9 months of age, White infants with limited other-race exposure show a reduced ability to identify individuals that are Asian^14^. Finally, Asian eyes stimuli used in the current study are controlled for a number of perceptual confounds (eye whites, iris, and pupil size), to further ensure that infants are responding to relatively subtle racial differences and not to coarse perceptual differences when processing own-race and other-race eyes.

Based on previous research that has shown that there is an enhanced activation in the STC during pupil mimicry in adults^18,24^ and work that has shown that behaviorally, infants only respond to pupil dilation (and not pupil constriction)^7^, we hypothesized that infants would show enhanced STC responses to pupil dilation^24^. Moreover, given the behavioral findings that pupil dilation mimicry is greatest for own race faces^11,31^, we hypothesized that STC activation to pupil dilation may be limited to, or at least enhanced when, viewing own-race eyes dilate.

Finally, we also examined whether, similar to adults, infants recruit frontal brain regions linked to cognitive control (dlPFC) when processing other-race eyes. Examining this in infants can inform the question as to whether such an effect on cognitive control exists in the human brain well before children display an overt social preference for own-race members^16^. If this were the case then this would indicate that dlPFC recruitment is the result of being unfamiliar with other-race faces rather than having overt racial (dis)preferences/biases^27,28,32^.

## Results

### fNIRS Analysis

To analyze our fNIRS data we conducted an omnibus repeated measures ANOVA with brain region (STC, dlPFC), hemisphere (left, right), race (own, other), and pupil (constriction, dilation) as within-subject factors. This analysis revealed a four-way interaction between brain region, hemisphere, race, and pupil, *F*(1, 26) = 4.93, *p* = 0.035, η^2^ = 0.16. To follow up on this interaction, which suggests that the pupil and race factors of interest interact with brain region, we carried out repeated measures ANOVAs for the two brain regions separately.

### STC

**Figure 1:**
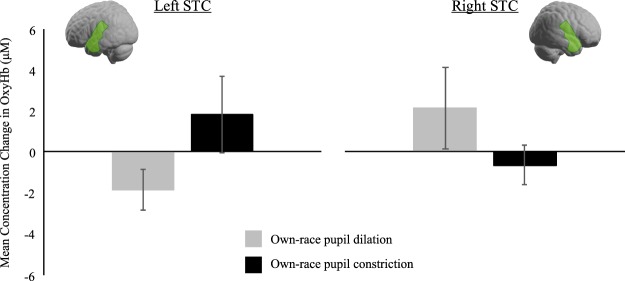
This shows the mean concentration changes in oxy-Hb in the left and right STC in response to own-race pupil dilation and constriction and the approximate cortical location. Note, error bars indicate standard errors.

For the STC region, we conducted a repeated measures ANOVA with hemisphere (left, right), race (own, other), and pupil (constriction, dilation) as within-subject factors. This analysis did not reveal any significant effects (all *p*-values > 0.077). Although we failed to find an effect in this analysis, we still decided to conduct separate repeated measures ANOVAs for own-race and other-race eyes in order to test our hypothesis that infants’ sensitivity to pupillary changes in others is affected by race and might be more pronounced for own-race eyes. In this analysis, we obtained a significant interaction between hemisphere and pupil only in the own-race context, *F*(1, 26) = 4.43, *p* = 0.045, η^2^ = 0.15. Specifically, own-race pupil dilation evoked greater responses than own-race constriction in the right hemisphere (own-race dilation: *M* = 2.12 μM, *SE* = 1.99, own-race constriction: *M* = −0.68 μM, *SE* = 0.96), whereas the reverse pattern was seen in the left hemisphere, where own-race constriction evoked greater responses than own-race dilation (own-race dilation: *M* = −1.84 μM, *SE* = 0.97, own-race constriction: *M* = 1.84 μM, *SE* = 1.86; see Fig. 1). For the other-race context, there were no significant effects (all *p-*values > 0.19).

We also assessed whether STC responses were impacted by other-race experience. For this purpose, we conducted an additional repeated-measures ANOVA with STC responses to own-race pupillary changes (dilating, constriction) and hemisphere (left, right) as a within-subjects factors and other-race experience (at least once a week, less than three times a month) as a between-subjects factor. This analysis revealed that other-race experience did not significantly impact STC responses (*p*-values > 0.22).

### dlPFC

**Figure 2:**
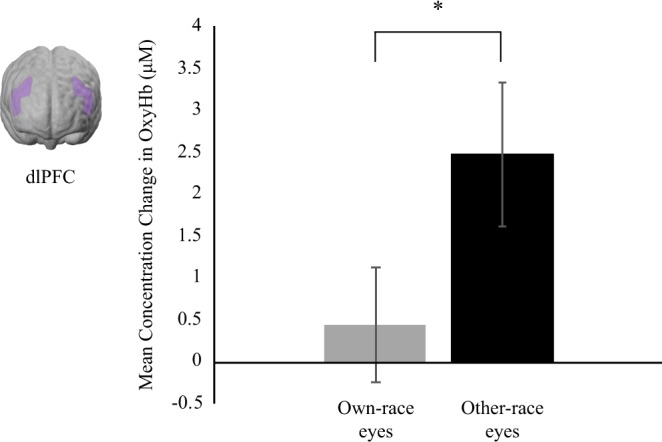
This shows the mean concentration changes in oxy-Hb in the dlPFC in response to own-race and other-race eyes and the approximate cortical location. Note, ^*^indicates *p*-value < 0.05 and error bars indicate standard errors.

For the dlPFC region, we conducted a repeated measures ANOVA with hemisphere (left, right), race (own, other), and pupil (constriction, dilation) as within-subject factors. Our analysis revealed a main effect of race on brain responses in the dlPFC region, *F*(1, 26) = 6.26, *p* = 0.019, η^2^ = 0.19, with other-race eye stimuli evoking greater responses (*M* = 2.48 μM, *SE* = 0.86) than own-race eye stimuli (*M* = 0.44, μM, *SE* = 0.69; see Fig. 2). There were no other main effects or interactions (*p* > 0.068)

Similar to as we reported above for the STC analyses, we also assessed whether differential brain responses in the dlPFC were affected by other-race experience. To do this, we conducted an additional repeated measures ANOVA with dlPFC response to race (own, other) as a within-subjects factor and other-race experience (at least once a week, less than three times a month) as a between-subjects factor. This analysis showed that other-race experience did not significantly impact dlPFC responses to own- and other-race eyes (all *p*-values > 0.20).

### Temporal Parietal Cortex

As an additional analysis of the fNIRS data, a repeated measures ANOVA with hemisphere (left, right), race (own, other), and pupil (constriction, dilation) as within-subject factors was conducted in an additional brain region, the temporal parietal cortex (TPC). This brain region was selected because prior work has identified that the TPC is sensitive to information regarding mental states^33,34^. However, this analysis did not reveal any significant effects (all *p-*values > 0.16).

Furthermore, in each of the three regions of interest (dlPFC, STC and TPC) we assessed effects on deoxy-Hb concentration changes across experimental conditions. This analysis revealed no significant effects for the deoxy-Hb concentration changes (all *p*-values > 0.10; see supplemental materials for further information). The absence of deoxy-Hb effects is in agreement with a number of infant fNIRS studies that also did not observe condition effects on deoxy-Hb^35^.

### Looking Time Analyses

**Figure 3:**
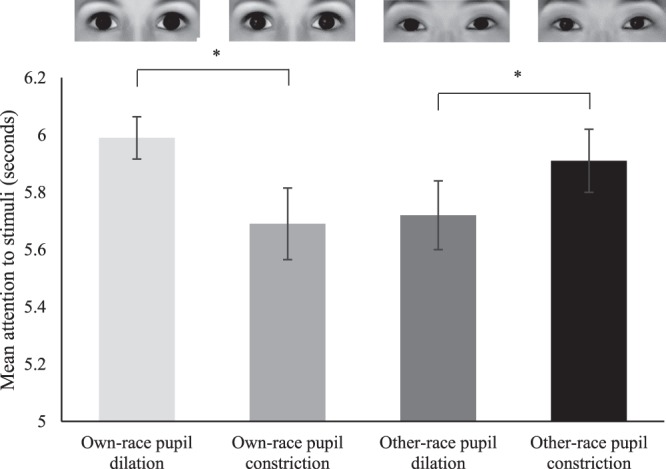
This shows the mean looking time to pupillary changes (dilation and constriction) for both own-race and other-race eyes. Please note that infants viewed photographic images of real eyes (see Methods) and that the eye images shown here were computer generated with the FaceGen software (https://facegen.com) for illustrative purposes. Note, ^*^indicates *p*-values < 0.05 and error bars indicate standard errors.

In addition to our primary analysis of the fNIRS data we carried out an analysis of looking time (duration of attention to the eye stimuli) coded from video. Specifically, we conducted a repeated measures ANOVA with race (own-race eyes, other-race eyes) and pupillary change (dilating, constricting) as within-subjects factors, to assess whether infants displayed any systematic differences in looking time (duration of attention/fixation on eye stimuli). This analysis revealed a significant interaction between the factors race and pupillary change, F(1, 26) = 9.64, *p* = 0.005, η^2^ = 0.27. There were no main effects of race or pupillary change (*p*-values > 0.48). A follow-up analysis performed by using paired-samples *t-*tests separately for own- and other-race eyes revealed that for own-race eyes, infants looked significantly longer at pupil dilation (*M* = 5.99 seconds, *SE* = 0.07) than at pupil constriction (*M* = 5.69 seconds, *SE* = 0.13), *t*(26) = −2.40, *p* = 0.024. In contrast, for other-race eyes, infants looked significantly longer to pupil constriction (*M* = 5.91 seconds, *SE* = 0.11) than to pupil dilation (*M* = 5.72 seconds, *SE* = 0.12), *t*(26) = 2.32, *p* = 0.028 (see Fig. 3).

Considering the obtained looking time differences reported above, we carried out an additional correlation analysis to examine potential associations between looking time and brain responses measured by fNIRS. In particular, given our a priori hypothesis about the right STC and the pattern of the observed response that suggests that the right STC response was enhanced to own-race pupil dilation when compared to own-race constriction, we conducted a Pearson’s correlation between right STC to own-race dilating eyes and looking time to own-race dilating eyes to test if the STC response observed was impacted by attentional processes. However, this analysis showed that looking time and right STC response to own-race dilating eyes were not significantly correlated (r(26) = 0.09, p = 0.66). This indicates that the looking time increase is unlikely to account for the enhanced brain response seen to dilating own-race eyes.

## Discussion

The current study investigated the neural basis of processing others’ pupillary changes and how race impacts these processes in infancy. We measured 9-month-old infants’ brain responses to dilating and constricting pupils in the context of viewing own-race and other-race eyes. Our fNIRS results show that only when responding to own-race eyes, infants’ brains distinguished between dilating and constricting pupils. Specifically, infants showed enhanced responses in the right superior temporal cortex when observing own-race pupil dilation and in the left superior temporal cortex when observing own-race pupil constriction. Our finding of a pupil change-sensitive brain response localized to superior temporal brain regions in infants is in line with previous work with adults^18,19,36,37^, suggesting the early ontogenetic emergence of brain function related to processing pupillary change cues. Furthermore, our results add to a growing body of research with infants, demonstrating that superior temporal brain regions are critically involved in processing social information from faces including eye gaze cues^20,22^.

It is important to emphasize that our infant data are the first to show that superior temporal cortex responses to others’ pupillary changes are limited to own-race stimuli, because previous fMRI research with adults only examined brain responses in an own-race context^18,19^. However, this finding is in line with previous fMRI work with adults, demonstrating that the amygdala is only sensitive to pupillary signals in the context of own-species faces but not in the context of other-species (cat) faces^37^. Moreover, the current finding with infants is in agreement with behavioral work with adults, showing that pupil mimicry as well as trust decisions are modulated by race^11,31^. The obtained absence of a discriminatory brain response in the other-race context concurs with previous research with infants of similar ages, showing that face identity and emotion discrimination is impaired when other-race stimuli are employed^12–14^. It is thus possible that the current results, in conjunction with previous work, index a more general impairment of infants’ social perceptual processes in the context of other-race faces.

However, it should be mentioned that our analysis of infants’ looking time responses indicates that infants are able to discriminate between pupil dilation and constriction regardless of racial context, because there are significant differences in looking time between dilation and constriction for both other-race and own-race eyes. Still, while our looking time results show that discrimination of pupillary change occurs in both racial contexts, the observed effects on looking time depend on racial context. Specifically, for own-race eyes infants displayed an increase in looking time to pupil dilation, whereas for other-race eyes infants showed an increase in looking time to pupil constriction. This pattern of looking time effects is interesting in the light of research showing that large pupils tend to be perceived as more positive, whereas small pupils tend to be perceived as more negative or even angry^4,6,19,36–38^. It may thus be possible that larger pupils receive heightened attention from infants when seen during an interaction with an own-race (in-group) member because it is a potentially positive signal, whereas smaller pupils receive heightened attention during an interaction with an other-race member because it is a potentially negative or threatening signal. However, it should be noted that previous work has not found differences in looking time to eyes when viewing partners with dilating as compared to constricting pupils; therefore, this rather speculative proposal should be thoroughly investigated in future work by manipulating the facial expressions of the person displaying changes in pupil size^39^. In any case, the analysis of the looking time data, which was not the primary focus of the current fNIRS study, provides behavioral evidence that race impacts the processing of pupillary changes in infants.

Though these results using looking time provide additional behavioral insights into how race impacts the processing of pupillary changes, one vital question remains. Namely, does infants’ superior temporal cortex response when viewing pupillary changes, especially during dilation, correlate with infants’ own pupillary response in the sense of pupil dilation mimicry as seen in prior infant work using pupillometry? Considering that we did not concurrently measure infants’ pupillary changes when viewing the eye stimuli in our study, we could not directly assess pupil mimicry and its neural correlate in our infant sample. However, there is fMRI work with adults showing that STC activity correlates with one’s own pupillary changes^18^, suggesting that such a link may exist. It is therefore possible that infants’ observed enhanced response in STC when viewing own-race pupil dilation may reflect brain processes linked to pupil mimicry. In this context, it is also important to mention that enhanced brain response seen in the right STC did not correlated with the heightened looking time observed in response to own-race pupil dilation. Thus, though it is possible that the STC response is associated with pupillary changes in the infant observer, the STC response is not linked to looking time.

Our results further show that infants recruit the dorsolateral prefrontal cortex, a brain region linked to cognitive control functions, when processing other-race pupillary changes. This is in line with previous fMRI research with adults and may suggest that interracial interactions afford greater cognitive control or effort from the infants^26^. This finding may inform a debate in the adult literature regarding the nature of dlPFC involvement during interracial contact, which could either be construed as the result of being unfamiliar with other-race faces or from the development of a racial bias or stereotype that requires cognitive control^26–28^. The current evidence for dlPFC involvement during interracial interactions in infants appears to favor the unfamiliarity (or novelty) view because, as outlined in the introduction, infants at this age do not display any overt behavioral preferences for own-race members or (dis-)preferences for other-race members; and, it is unlikely that infants have acquired adult-like racial stereotypes they need to suppress through exerting cognitive control during interracial contact^40^. Previous fNIRS research with infants shows that presenting novel stimuli results in enhanced dlPFC responses^30^, tentatively supporting the interpretation that enhanced dlPFC responses are related to infants being unfamiliar and likely lacking face-to-face interaction experience with individuals from the other-race. Nevertheless, it is possible that the recruitment of dlPFC during interracial interactions is initially a result of being unfamiliar with another-race individual, but later develops into reflecting the suppression of racial stereotypes. Clearly, more research across a wider age range is required to test the development of dlPFC function during interracial interactions.

At this point it is important to mention that experience with other-race individuals as measured through parental report did not have an effect on infants’ brain responses in the current study. Previous research has reported that infants growing up in the urban United States spend on average 92% of their face-to-face interactions with own-race individuals (see Rennels & Davis, 2008). It is thus possible that even though 55.5% of parents in the current study reported that their infants had some exposure to other-race individuals these interactions may not have been long or engaging enough to facilitate infants’ processing of information from other-race faces.

One promising approach to further test the nature of dlPFC involvement and cognitive control during interracial interactions, would be to carry out training studies with infants. In fact, novel training studies, such as exposing infants to books that identify individuals from other-races, are effective strategies to improve the recognition of other-race individuals^40^. It will be important to examine whether this kind of training transfers to the current context and whether reduced dlPFC recruitment may be seen after the training. Moreover, with respect to the role of experience, it will be critical to extend the current research to Asian (Japanese) infants in order to examine whether the effects seen in the current study generalize across race. Previous research using the same stimuli has found similar patterns of perceptual narrowing across Asian and White infants; such that, White and Asian infants were able to recognize facial identities of other-race faces at 6 months of age (e.g., Asian infants were able to recognize both White and Asian faces) but by 9 months of age both sets of infants were only able to identify own-race faces^14,41^. Given the previous work, it would be interesting to test if Asian infants show the same pattern of results (e.g., recruit dlPFC in response to White eyes and are able to distinguish pupillary cues only in the context of Asian eyes).

In conclusion, the current study identifies the superior temporal cortex as a brain region involved in infants’ processing of pupillary changes from others. Our data further demonstrate that, early in development, brain processes of responding to pupillary changes in others are impacted by race and that inter-racial eye-based interactions may afford greater cognitive control or effort from infants, perhaps due to their novelty. This critically informs our understanding of the neurodevelopmental origins of pupil mimicry and highlights the impact that group-membership cues such as race have on processing social information from the eyes.

## Method

Twenty-seven infants (14 girls, 13 boys; *M* [age] = 9 months, 23 days; ranging from 9 months, 3 days to 10 months, 21 days) were included in the final sample used for analysis. All of the participants were White. For the present study, we administered a parental report measure of other-race exposure to assess infants experiences with other-race individuals and the majority of infants had minimal experience with any other-race individuals (55.6% interacted with other-race individuals more than once per week; 44.4% interacted with other-race individuals less than 1–3 times per month). All participants were born at term, with normal birth weight (>2,500 g), and did not have any hearing or visual impairments. Nineteen additional infants were tested but were excluded from the present analyses. Note that this attrition rate is similar to previous infant fNIRS studies employing three or more experimental conditions^42–44^. Out of the 19 excluded infants, most were excluded because they failed to reach our pre-determined looking criterion of attending to the visual presentation on the screen for a minimum of 60% of the trial (4.2 s.) for at least two trials per condition (*n* = 13) and the others were excluded for being fussy or upset throughout the experiment (*n* = 6). Participants were recruited from a large database of infants and children in a mid-sized college town in the mid-Atlantic region of the US. All parents gave informed consent for their infants to participate in accordance with the Declaration of Helsinki and infants received a small toy for their participation. All procedures were approved by and carried out in accordance with The University of Virginia Institutional Review Board for Social and Behavioral Sciences.

### Stimuli

Stimuli were adapted from previous behavioral studies of pupil mimicry in adults^11,31^. These stimuli consisted of images of the eye regions cropped from full-face neutral expression White (own-race) and East Asian (other-race) actors, onto which dynamically changing pupils were superimposed (please refer to the following papers for example stimuli: Kret, Fischer & De Dreu, 2015; Kret & De Dreu, 2017). Note that a series of controls were put in place by Kret and colleagues in order to reduce possible perceptual confounds: (1) both own-race and other-race eyes were identical for the inner features of the eyes (eye white, iris, and pupil), (2) the stimuli were presented as gray-scale in order to reduce perceptual differences due to contrasting skin tone and eye color, and (3) although there were individual differences across actors for eye shape, previous analyses show that there is no statistical difference in the amount of eye white visible across own-race and other-race eyes^11^.

**Figure 4:**
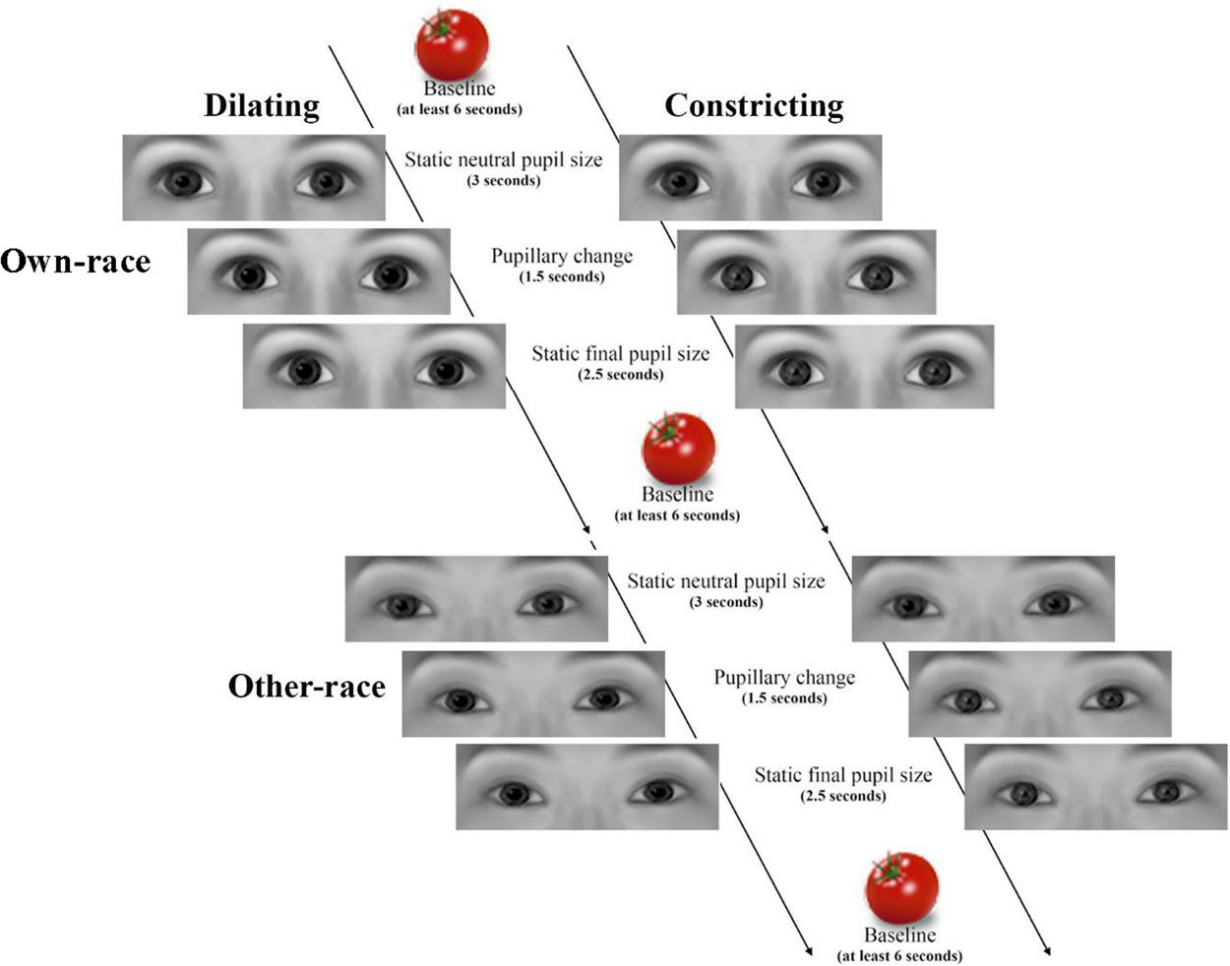
Illustration of the experimental procedure used in the current study. Please note that infants viewed photographic images of real eyes (see Methods) and that the eye images shown here were computer generated with the FaceGen software (https://facegen.com) for illustrative purposes.

Each pupillary stimulus presentation consisted of three stages: (1) starting with a static neutral sized pupil (5 mm) presented for 3 seconds, (2) followed by a dynamic pupil size change, which could either be a dilation (going from 5 to 7 mm) or a constriction (going from 5 to 3 mm), presented over the course of 1.5 seconds, (3) followed by a static pupil, in either the fully dilated (7 mm) or the constricted (3 mm) state, presented over 2.5 seconds (see Fig. 4 for an overview of the experimental paradigm). Please note, the timing for the experimental stimulus presentation is longer in this experiment compared to previous eye-tracking experiments that have used the same stimuli^11^,^24^,^31^. This adjustment was made in order to account for the delay in the hemodynamic response measured by fNIRS. Pupil sizes were selected to reflect the normal range of pupillary fluctuation^11,31^. Five different inanimate objects (vegetables) were used as the inter-stimulus interval images. These images have been used in previously published fNIRS studies examining facial processing in infancy^23,45,46^ and have served as an established non-social inter-stimulus interval. Note that while a non-social inter-stimulus interval was employed and any changes in infants’ brain responses reflect a change from the non-social stimulation, in line with previous studies, we did not employ a baseline subtraction method whereby the concentration changes measured during the non-social inter-stimulus interval were subtracted from the four different eye stimulus conditions. A shaking rattle video clip (from Tobii software, Sweden) and accompanying audio (three tones ranging from 109 Hz to 262 Hz) was presented after four experimental trials. This was used to regain infants’ attention and orient them to the center of the screen. The videos were 500 pixels wide and 300 pixels high, resulting in an eye region which is 13.5 cm wide and 6 cm high on the computer screen. Please note, the sizes of the stimuli and pupillary changes were selected based on prior work that has been successful in eliciting differential behavioral responses in pupil mimicry for similar age infants^7^.

### Procedure

Infants sat on their parents’ laps in a quiet, dimly lit room, at a distance of approximately 60 cm from the screen (23-inch monitor). The distance of the infant from the screen and size of the eye stimuli were selected to reflect the experience of a close-range face-to-face interaction^11^. A small plastic teething ring was available for each infant to hold during the study to increase attentiveness to the stimuli and decrease bodily movements during the experiment^47,48^.

The experimental paradigm was presented by using the Presentation software package (Neurobehavioral Systems, USA). Infants were presented with a total of 32 eye stimuli (8 own-race/constricting, 8 other-race/constricting, 8 own-race/dilating, 8 other-race/dilating). Each experimental trial lasted seven seconds. Between each experimental trial, there was a brief bell sound (about 150 milliseconds and 600 Hz) and a dynamic non-social inter-stimulus interval (which was jittered and presented for at least six seconds)^23,45,46,49^. The sequence of the presentation was pseudo-randomized so that no more than two consecutive trials were from the same type of race (own or other) or pupillary change (dilation or constriction). To ensure that the infants looked at the screen, each experimental trial was started manually by the experimenter when the infant attended to the screen, resulting in variable inter-stimulus intervals. The entire experimental session took approximately 10 minutes.

### Data acquisition

Infants’ fNIRS data were recorded using a NIRx Nirscout system and NirStar acquisition software. The fNIRS method quantifies concentration changes of oxygenated hemoglobin (oxy-Hb) and deoxygenated hemoglobin (deoxy-Hb) in the cerebral cortex. This is done by utilizing specific frequencies of near-infrared light that are selectively absorbed by oxy-Hb and deoxy-Hb (for an overview see^50^). The NIRx Nirscout system used contains 16 source-detector pairs, arranged to result in a total of 49 channels (see Supplemental Materials). A fabric cap (Easycap) configured the source-detector pairs approximately 2.5 centimeters apart in the frontal and temporal cortices in both hemispheres. Data were recorded at a sampling rate of 3.91 Hz. Near-infrared light-emitting diodes were emitted at two wavelengths at 780 nm and 830 nm with a power of 20 mW/wavelength. The fNIRS system automatically made adjustments for light intensity in order to provide optimal gain. A camera mounted above the screen recorded infants’ behavior during the experiment and allowed for later coding of attention throughout the experiment.

### Data analysis

Infants’ attention during the fNIRS paradigm was coded offline by a trained research assistant from video recordings of the experimental session. Specifically, the time the infant spent looking at each experimental trial was recorded. To assess the reliability of the attentional coding done by the primary coder, an additional trained coder also coded infant looking time from a randomly selected subsample of infants (25.9%; *n* = 7). This analysis showed that inter-rater reliability was excellent (Cronbach’s α = 0.94). Note, trials were only included if the infant looked for more than 60% of the presentation time of an individual experimental trial (4.2 seconds). For each infant, the average looking time across all included trials for each experimental condition was calculated and used as a measure of attention to stimuli in the Results section.

In addition, all trials were excluded that exhibited motion artifacts (determined by visual inspection of the hemodynamic response across all individual channels). In order for infants to be included in our data analysis, they had to contribute at least two artifact free trials with adequate attentiveness per condition (own-race/dilating, own-race/constricting, other-race/dilating, other-race/constricting). This threshold of two artifact free trials for which infants looked for at least 60% of the time is similar to previous fNIRS research with infants^51^ and all trials that did not reach this threshold were removed prior to data analysis. The final sample included 27 infants that on average contributed data for a total of 17.19 trials, *SD* = 6.77 (*M* trials per condition = 4.30, *SD* = 1.69). Note, we conducted a repeated measures ANOVA to assess if there were any systematic differences in attention to trials across conditions and we found no significant differences in the number of trials included across conditions (*p* = 0.91).

The fNIRS data were analyzed using the Matlab-based software nilab2 (see the following papers for other fNIRS data analyzed using this software)^52,53^. Data were filtered using a 0.2 Hz low-pass filter (to remove fast fluctuations related to heart rate) and a high-pass filter of 0.07 Hz (to remove changes that were too slow and related to drift). Oxy-Hb and deoxy-Hb concentration changes were calculated for each condition using the modified Beer-Lambert law and baseline corrected (whereby all the experimental hemodynamic responses are set to begin at 0 mM with stimulus onset). We computed hemodynamic concentration changes in response to the stimulus conditions. The stimulus length was set to 7 seconds (which reflects the stimulus duration from eyes onset to eyes offset). The boxcar functions corresponding to the four stimulus conditions were convolved with a standard hemodynamic response function based on the stimulus length parameter^54^. In line with previous reports from vascular imaging in infants with the BOLD contrast and optical topography^55,56^, we used parameters similar to those used in adult subjects, assuming a peak response at 5 s (τ = 1). The analysis time window considered for computing concentration changes was set from 0 (stimulus onset) to 20 seconds (which takes into account this lag in the hemodynamic response peak).

**Figure 5:**
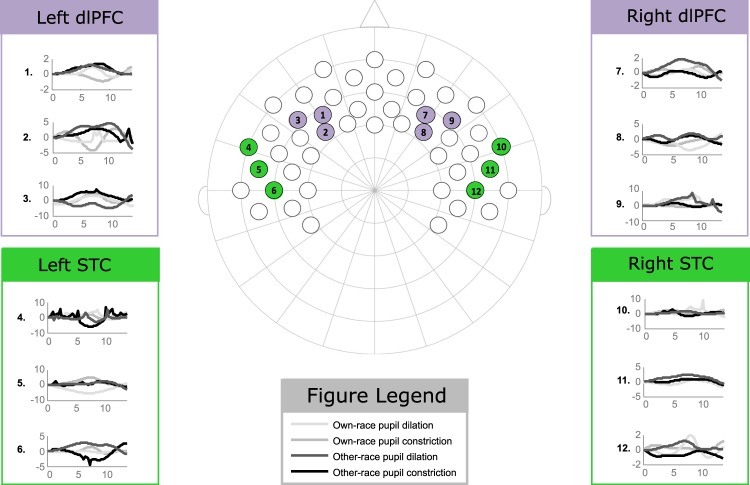
This shows the fNIRS channel placement with respect to the relevant 10–20 system and the associated oxy-Hb hemodynamic response (the units for the y-axis are in μM) across each experimental condition (the units for the x-axis are seconds). The ROIs are color-coded as follows: dlPFC (purple), STC (green).

Analyses examining condition differences were conducted for both oxy-Hb and deoxy-Hb changes. Furthermore, regions of interest were created for the STC and the dlPFC in both hemispheres; these regions were based on previously published information regarding the cortical projection of the 10–20 EEG system^57^ and carried out by selecting the corresponding fNIRS channels (see Fig. 5). The channels selected for the STC approximately corresponded with the FT8 and C6 electrode positions in the right hemisphere and FT7 and C5 electrode positions in the left hemisphere. The channels selected for the dlPFC approximately corresponded with the F4 and F6 electrode positions in the right hemisphere and the F3 and F5 electrode positions in the left hemisphere. Lastly, the channels selected for the control region, the TPC, approximately corresponded with the CP4 electrode position in the right hemisphere and the CP3 electrode position in the left hemisphere.

## Supplementary information

Supplementary Materials

## Data Availability

For more information on study stimuli please contact MK. The dataset for the present study is available from the corresponding author (TG) at request.
