# Supplementary material for: Infants’ brain responses to pupillary changes in others are affected by race
Source: Sci Rep. 2019 Mar 13;9:4317. doi: 10.1038/s41598-019-40661-z (PMC6416351; doi:10.1038/s41598-019-40661-z)
Supplement: Supplementary file 1 — Supplementary Materials [file 41598_2019_40661_MOESM1_ESM.docx]

Infants’ brain responses to pupillary changes in others are affected by race

Supplemental Materials

Caroline M. Kelsey^1^, Kathleen M. Krol^1^, Mariska Kret^2,3^, & Tobias Grossmann^1,4*^

^1^ Department of Psychology, University of Virginia, Charlottesville, VA, USA.

^2^ Institute of Psychology, Cognitive Psychology Unit, Leiden University, Leiden, The Netherlands

^2^ Leiden Institute for Brain and Cognition (LIBC), Leiden University, Leiden, The Netherlands

^4^ Max Planck Institute for Human Cognitive and Brain Sciences, Leipzig, Germany

^*^Correspondence concerning this article should be addressed to:

Tobias Grossmann

Department of Psychology

PO BOX 400400

University of Virginia

Charlottesville, VA 22904

grossmann@virginia.edu

Right STC

Left STC

Own-race pupil constriction

Own-race pupil dilation

*Supplementary Figure 1.* This shows the mean concentration changes in deoxy-Hb in the left and right STC in response to own-race pupil dilation and constriction.

Own-race eyes

Other-race eyes

*Supplementary Figure 2*. This shows the mean concentration changes in deoxy-Hb in the dlPFC in response to own-race and other-race eyes.
